# The Clinical Caveat for Treating Persistent Hypokalemia in Diabetic Ketoacidosis

**DOI:** 10.7759/cureus.42272

**Published:** 2023-07-21

**Authors:** Bashar Khiatah, Amanda Frugoli, Deborah Carlson

**Affiliations:** 1 Internal Medicine, Overlake Medical Center, Bellevue, USA; 2 Pacific Inpatient Physicians, Community Memorial Hospital, Ventura, USA; 3 Graduate Medical Education, Community Memorial Hospital, Ventura, USA; 4 Internal Medicine, Community Memorial Hospital, Ventura, USA

**Keywords:** keto acidosis, current guidelines, insulin treatment, diabetic ketoacidosis (dka), refractory hypokalemia

## Abstract

The medical community seeks to provide evidence-based guidelines for treating any disease to ensure optimal care delivery. Occasionally, a patient's unique physiology does not respond to guideline-driven treatments and requires experienced clinical personalization for treatment. Failure of clinicians to recognize patient outliers and augment care can delay treatment, provide substandard care, and potentially threaten a patient's life. This paper describes a clinical caveat for treating profound or persistent hypokalemia in patients with DKA (diabetic ketoacidosis).

## Introduction

Diabetic ketoacidosis (DKA) is one of the most common causes of emergency visits in patients with diabetes mellitus. Diabetic ketoacidosis is a relative or absolute insulin deficiency complication with a concurrent elevation of counter-regulatory hormones such as cortisol, catecholamines, growth hormone, and glucagon. The result is the classic triad of hyperglycemia, high anion-gap metabolic acidosis, and ketosis, which are often accompanied by variable degrees of electrolyte abnormality, specifically potassium volume and depletion. Diabetic ketoacidosis occurs mainly in patients with uncontrolled type 1 diabetes mellitus and in adults with poorly controlled type 2 diabetes mellitus (T2DM) due to impaired insulin secretion or action under stressful circumstances, such as acute illnesses, medical or surgical, and, in adolescents, in new-onset T2DM. Although any illness or physiological stress can precipitate DKA, the most frequent causes are medication noncompliance, infections, particularly urinary tract infections, and gastroenteritis [1,2]. 

## Technical report

Pathophysiology

**Figure 1:**
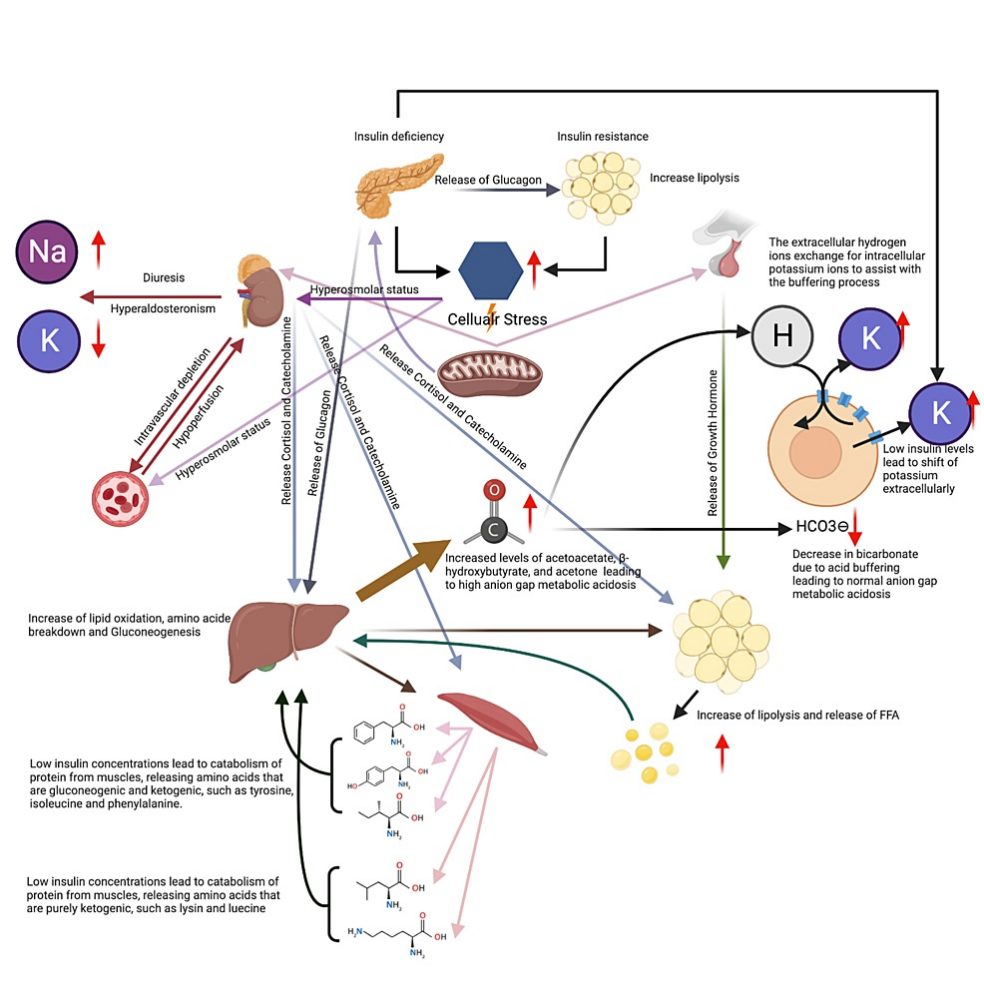
DKA pathophysiology This work is original and created by Dr. B. Khiatah using BioRender.com.

Changes in counter-regulatory hormone levels and insulin deficiency or reduction in its activity lead to alterations in glucose production, excretion, increased lipolysis, and ketone production through gluconeogenesis. Low insulin concentrations will also lead to the catabolism of muscle protein-releasing amino acids that are gluconeogenic and ketogenic and the formation of acetyl coenzyme A (acetyl-CoA) and β-hydroxy-β-methylglutaryl-CoA (HMG-CoA), all of which result in ketone body production [3,4]. Elevated glucagon, catecholamine, and cortisol levels stimulate gluconeogenic enzyme activity, which augments (accelerates) hyperglycemia. Among these enzymes are phosphoenolpyruvate carboxykinase, pyruvate carboxylase, and fructose-1,6-bisphosphatase [4]. Both insulin deficiency and counter-regulating hormones activate hormone-sensitive lipase in adipose tissue, inducing lipolysis. The induction of lipolysis releases a large amount of free fatty acids (FFAs) with high glucagon concentrations that are oxidized in the liver by mitochondria, producing ketone bodies such as acetoacetate, β-hydroxybutyrate, and acetone. These compounds are responsible for the classic 'fruity' breath in patients with DKA [3] (Figure 1).

Acidosis results from proton buffering produced by the degradation of keto acids at physiological pH. The reduced clearance of ketones, which remains unknown, contributes to the high anion circulation concentration [3,5]. The accumulation of ketoacids drives a decrease in serum bicarbonate levels and the development of high anion gap metabolic acidosis. Measurement of blood pH is essential because intracellular biological systems begin to fail as pH levels fall below 7.35. As acidosis progresses, the impact becomes more severe, with irreversible damage at pH <6.8, resulting in neurological manifestations including confusion, coma, and possible death [5]. The accumulation of glucose, acetoacetate, and β-hydroxybutyrate results in osmotic diuresis, leading to hypovolemia and generalized hypoperfusion, which generate lactic acid and decrease the glomerular filtration rate, thereby reducing glucose excretion and worsening the already hyperglycemic state. Together, these factors cause prerenal failure, leading to inadequate excretion of acids such as sulfate, phosphate, or urate, further exacerbating high anion gap acidosis. Vomiting associated with osmotic diuresis or a lower consciousness level affecting the ability to take fluids orally can also worsen dehydration [2,6-7].

Potassium 

Potassium, a predominantly intracellular cation, is maintained at normal levels intracellularly by insulin. Thus, insulin deficiency results in potassium shifting to the extracellular space. As mentioned earlier, when the pH decreases further, the bicarbonate ion concentration drops to buffer the increase in hydrogen ion concentration. This buffering mechanism is a crucial compensatory mechanism to prevent the rapid denaturation of proteins from rapid changes in pH. Another mechanism to preserve physiologic pH includes ion exchange between extracellular hydrogen ions and intracellular potassium ions [8]. These physiological efforts to maintain buffering capacity and electrical neutrality are called hyperkalemia [9]. It has been reported that, for each 0.1 unit fall in pH, the serum potassium concentration increased by 0.6 mM/l [9]. That being said, in the acute state prior to fluid and insulin treatment, serum potassium can be as high as ≥7.0 mM/l; however, because of the renal loss, total body potassium is usually substantially depleted [2,8] The cellular shifts result in extracellular hypertonicity, leading water to diffuse from the intracellular space to the extracellular space. In addition to this shift, the osmotic diuresis and the circulating volume decrease (vomiting or low consciousness level), resulting in decreased circulating volume and activating a secondary aldosterone level increase. Increases in aldosterone, a mineralocorticoid, stimulate sodium reabsorption in the distal tubules of the kidney using the epithelial sodium channel (ENaC). Aldosterone also activates Na+/K+ exchange pumps, which results in the reabsorption of sodium (Na+) ions into the blood and the secretion of potassium (K+) ions into the urine, which leads to increased potassium loss [8,9]. Almost all patients with DKA have a substantial potassium deficit on presentation, and potassium replacement is almost always required [2]. The resolution of DKA begins as soon as insulin treatment starts, as it suppresses ketone production and resolves acidosis. On the other hand, insulin shifts potassium back intracellularly, and patients might develop profound hypokalemia that occurs despite aggressive replacement [10-11]. Thus, frequent potassium monitoring during the first few hours of treatment is crucial. Because this potential rapid shift in potassium levels concerns the development of cardiac arrhythmias, continuous cardiac monitoring is recommended [12]. Severe hypokalemia in patients with DKA has been associated with an increased mortality rate [11]. Therefore, it is recommended to stop insulin administration for any patient who develops symptomatic hypokalemia until the potassium level is above 3.3 mM/L. The American Diabetes Association, the American Association of Clinical Endocrinology, and other references have recommended initiating insulin infusion until the potassium level is > 3.3 mM/L [13-15]. On the other hand, another European reference recommended starting the insulin infusion rate at 0.05 units per kilogram per hour [16].

Question

What happens when a young patient presents after several days of new-onset symptoms (polydipsia, polyuria, nausea, and vomiting) and is hypovolemic, in moderate acidosis, and profoundly hypokalemic (K<3.3 mm/l)?

## Discussion

According to a study conducted at the University of Southern California, the prevalence of hypokalemia in patients with DKA is 5.6% [16]. It has been a clinical challenge to treat DKA patients with profound refractory hypokalemia. With the current guideline advising against initiating insulin therapy due to fear of cardiac arrhythmia, many patients face another life-threatening acidosis, cardiac arrhythmia due to hypokalemia, and severe neural complications. Few case reports have been published to address these critical subjects, with different therapeutic approaches such as continuous renal replacement therapy, hemodialysis, and the initiation of insulin infusion at a low rate [17-18]. 

Technically, to treat hypokalemia appropriately, we need to replace fluids that will increase the volume, decrease aldosterone activity, stop potassium loss through urine, and stop ongoing ketosis by starting insulin. That will shift glucose intracellularly and reduce ketone body formation, which stops acidosis progression which will affect hydrogen potassium exchange at the cellular level. Diabetic ketoacidosis resolution criteria include a combination of a blood glucose concentration of <200 mg/dL, a pH of >7.30, a serum bicarbonate level of ≥18.0 mM/L, a calculated anion gap of ≤14.0 mmol/L, and in some references, a serum β-hydroxybutyrate level of <1.0 mM/L [13]. 

Point

After initiating aggressive fluid replacement, without stopping the culprit of the DKA process (insulin deficiency or insufficiency), ketosis, gluconeogenesis, potassium loss, and acidosis will continue. Replacing fluids will temporarily help hypovolemia but will not stop osmotic diuresis since gluconeogenesis does not stop with fluids. Potassium replacement will fill in what is being lost actively through the kidneys but will not correct or elevate the levels enough to start insulin placement according to the current therapeutic guidelines since it will be like pouring water in a strainer! We know that nothing in the human body's metabolism cycle is an on-off switch; thus, we understand that it takes time to correct acidosis, completely stop gluconeogenesis, clear counter-regulatory hormones, stop aldosterone secretion, and stop the formation of ketone bodies. That being said, we are pointing out the need to revisit the current guidelines for DKA therapy that recommend holding initiating insulin therapy until potassium levels are above 3.3 mM/L and initiating insulin at a lower rate after initiation of potassium replacement therapy orally and intravenously.

We acknowledge that this is only an opinion article laying out facts about current guidelines and shedding light on this matter that needs to be addressed, maybe by retrospective trials looking at delays in insulin treatment caused by an attempt to correct profound hypokalemic patients or even prospective trials that may result in an adjustment of current guidelines.

## Conclusions

Overall, we hope with this article to initiate a movement to revisit the current guidelines to be adjusted for this essential matter that physicians encounter on a regular basis across the country and to have more clear-cut guidance on this condition. We endorse that, in treating DKA patients with profound hypokalemia (K<3.3mM/L), it is essential to start enteral and parenteral potassium simultaneously with the initiation of insulin infusion at a lower rate to treat the culprit of hypokalemia, to treat DKA, and to avoid any insulin infusion delays in attempting to correct ongoing hypokalemia.
